# The effects of fermented feedstuff derived from Citri Sarcodactylis Fructus by-products on growth performance, intestinal digestive enzyme activity, nutrient utilization, meat quality, gut microbiota, and metabolites of broiler chicken

**DOI:** 10.3389/fvets.2023.1231996

**Published:** 2023-07-04

**Authors:** Xinhong Zhou, Huaidan Zhang, Shiyi Li, Yilong Jiang, Lijuan Kang, Jicheng Deng, Chuanpeng Yang, Xin Zhao, Jingjing Zhao, Li Jiang, Xianxin Chen

**Affiliations:** ^1^School of Life Science and Engineering, Southwest University of Science and Technology, Mianyang, Sichuan, China; ^2^Leshan Academy of Agriculture Science, Leshan, Sichuan, China; ^3^Leshan Animal Disease Prevention and Control Center, Leshan, Sichuan, China

**Keywords:** broiler chickens, fermented feed, growth performance, gut microbiota, gut microbiota metabolites

## Abstract

This research aimed to assess the impact of fermented Citri Sarcodactylis Fructus by-products (FCSF) on the growth performance, gut digestive enzyme activity, nutrient utilization efficiency, gut microbiota, and their metabolites in broiler chickens. A total of 1,080 male broiler chickens were allocated into four groups (T1–T4) consisting of 6 replicates per group, each containing 45 chickens. The basal diet was provided to group T1, while groups T2, T3, and T4 were supplemented with 1%, 3%, and 5% FCSF in the basal diet, respectively. The experimental period was 42 days. The findings revealed that supplementing FCSF improved the FW and ADG of broiler chickens, and led to a reduction in the F/G, ADFI, and mortality rate of broiler chickens (*p* < 0.05). Furthermore, supplementation with 3% and 5% FCSF improved the thigh yield, semi-eviscerated carcass yield, slaughter yield, and lipase activity in the duodenum and ileum of birds (*p* < 0.05). Additionally, supplementing 3% FCSF enhanced the activity of protease in the duodenum of broilers (*p* < 0.05). Moreover, supplementing 3% FCSF enhanced the utilization of total phosphorus, dry matter, crude protein, and crude ash in the feed by broilers (*p* < 0.05). Compared with the control group, supplementation of 3% and 5% FCSF reduced the serine content in broiler chicken breast meat (*p* < 0.05). Supplementing 1% FCSF significantly increased the C14:0, C14:1, and C20:1 content in the breast meat compared to the other experimental groups (*p* < 0.05). The levels of C20:4n6 and C23:0 in the breast meat of birds of FCSF supplemented groups were lower than in T1 (*p* < 0.05). Furthermore, the content of ∑ω-3PUFA decreased after supplementing with 3% and 5% FCSF (*p* < 0.05). 16SrDNA showed that supplementing 3% FCSF reduced the ACE, Chao1, and Shannon indices in the cecum of birds (*p* < 0.05). Supplementing 3% FCSF also decreased the abundance of the phylum *Desulfobacterota* and improved genera *Coprobacter* and *Prevotella* in the cecum of broiler chickens (*p* < 0.05). Metabolomic analysis of the gut microbiota revealed that supplementing 3% FCSF upregulated 6 metabolites and downregulated 16 metabolites (*p* < 0.05). Moreover, supplementing 3% FCSF downregulated 12 metabolic pathways and upregulated 3 metabolic pathways (*p* < 0.05). In summary our findings indicate that supplementing FCSF can improve the growth performance of broiler chickens by enhancing intestinal digestive enzyme activity, nutrient utilization, improving gut microbial diversity, and influencing the metabolism of gut microbiota.

## 1. Introduction

China produces a large amount of agricultural and industrial by-products every year (1). These by-products used to be treated as waste and caused pollution posing a threat to the environment in the past (2). With the societal development and technological advancements, there is a growing focus on transforming these by-products into valuable resources (3, 4). China holds a prominent position as a leading global producer of livestock, requiring a large amount of animal feed annually to sustain animal growth. The country heavily relies on imported feed ingredients to meet the demand, which leads to a growing competition between human and animal consumption (5). Thus, the advancement of novel feed resources plays a pivotal role in driving the progress of China's livestock industry. One of the solutions to the current feed shortage is the conversion of agricultural by-products or industrial waste into feed resources after deep processing (6).

In 2020, China banned the addition of antibiotic products in animal feed (7). However, the comprehensive ban on antibiotics has significantly influence on China's livestock industry, including increased production costs, increased difficulty in disease prevention and control, and decreased production (8). Nevertheless, this ban has also fostered the sustainable development of animal husbandry by reducing the presence of residual antibiotics in animals and mitigating the long-term risk of antibiotic resistance (9, 10). In the context of the comprehensive ban on antibiotics, there has been significant research and application of biological fermented feed, which can promote animal health by improving growth performance, enhancing immunity, improving intestinal health, and improving the quality of animal products (11–13). Therefore, the use of fermented feed can achieve partial or complete substitution of antibiotics in animal farming.

Citri Sarcodactylis Fructus is a medicinal plant originated from India and mainly cultivated in regions such as Guangdong, Sichuan, and Zhejiang in China. Modern medicine has shown that Citri Sarcodactylis Fructus has various effects, such as anti-depressant, anti-bacterial, anti-inflammatory, anti-cancer, anti-tumor, anti-aging, and blood pressure-lowering properties (14, 15). Citri Sarcodactylis Fructus is mainly used in deep processing for pharmaceutical development, food development, and cosmetics research. The byproducts of Citri Sarcodactylis Fructus in food processing and pharmaceutical industries are rich in high-quality nutrients such as protein, carbohydrates, and fats, which can be used as feed resources. However, they also contain high levels of crude fiber, lignin, and other anti-nutritional factors, which limit their direct use as animal feed. Research has shown that microbial fermentation can effectively reduce anti-nutrients and toxic substances in plant-based raw materials (16, 17). Therefore, we mixed Citri Sarcodactylis Fructus by-product with other raw materials in a certain proportion and subjected it to biological fermentation to produce a biological feed additive. As a dietary supplement for broiler chickens, we assessed its impact on growth performance, intestinal digestion function, intestinal microbiota, and metabolites of the broilers. The aim was to provide corresponding data support for the application and promotion of Citri Sarcodactylis Fructus by-product fermentation feed.

## 2. Materials and methods

### 2.1. Experimental design and animals

We randomly assigned 1,080 male cyan-shank partridge birds, aged 47 days, to four groups (T1–T4). Each group consisted of 6 replicate pens, housing 45 birds per pen. The pens were of dimensions 2.2 m in length, 2 m in width, and 1.2 m in height. The birds were raised for a period of 42 days. The basal diet was provided to group T1, while groups T2, T3, and T4 were supplemented with 1%, 3%, and 5% FCSF in the basal diet, respectively. All birds were obtained from a local commercial breeding farm and were reared under natural light with free access to water and feed provided twice daily. Feed remnants were collected and weighed half an hour after each feeding to calculate the average daily feed intake (ADFI). Daily mortality records were maintained for each replicate bird to calculate the mortality rate. At the end of the trial, the birds within each replicate pen were weighed to ascertain the average daily gain (ADG) and feed conversion ratio (F/G).

Metabolic experiments were conducted during the later stage of the trial. A 3-day pre-experiment was set up prior to the official trial, where one bird was randomly chosen from each replicate and placed in a metabolic cage for individual feeding. After a 3-day adaptation period, the chickens were subjected to a 48-h fasting period to allow the elimination of feces from their bodies. Subsequently, the chicken was fed continuously for 3 days, and its feces were collected for the following 5 days. The collected feces were sprayed with 10% sulfuric acid, and then dried in a 65°C oven to produce air-dried samples. The feed samples were prepared using the same method as the feces samples. The phosphorus, calcium, dry matter, crude ash, crude fat, and crude protein content in both the feed and feces were determined, and nutrient utilization was calculated.


$$\begin{array}{l} \text{ Apparent nutrient digestibility}\\ ={\left[\left(W1^{*}{W2-W3}^{*}W4\right)/{W1}^{*}W2\right]}^{*}\;100\% \end{array}$$

*W1*: feed intake, *W2*: nutrient content in feed, *W3*: fecal output, *W4*: nutrient content in feces.

### 2.2. Diets

In this experiment, we prepared two types of feed: a basal diet with its nutritional and compositional components shown in Table 1, and a fermented feed made of citri sarcodactylis fructus by-products with its compositional and nutritional components listed in Table 2. The citri sarcodactylis fructus by-products were obtained from a local production and processing cooperative. After they processed the citri sarcodactylis fructus, we collected the remaining by-products, dried, and crushed them, and used them as feed ingredients. The citri sarcodactylis fructus by-products have the following nutritional composition: crude ash content of 3.98%, crude protein content of 7.62%, crude fat content of 1.53%, crude fiber content of 12.27%, calcium content of 0.62%, and phosphorus content of 0.16%. We mixed the crushed citri sarcodactylis fructus by-products with other ingredients as listed in Table 2, placed them in a fermentation bag with a unidirectional respiratory valve, and fermented them for 3 days at 36°C. The fermentation was carried out using *Lactobacillus Plantarum, Saccharomyces cerevisiae*, and *Bacillus subtilis*, with their activity all >1^*^10^9^ CUF.

**Table 1:** The composition and nutritional level of the basic diet.

**Ingredients**	**Content (%)**	**Nutritional level^b^**	**Content (%)**
Corn	47.4	Metabolizable energy (MJ/kg)	12.83
Cottonseed meal	5	Crude protein	19.5
Chicken powder	4	Crude Ash	9.08
Corn bran residue	8	Moisture	11.25
Canola meal	3	Ca	0.73
Corn protein powder	4.9	P	0.37
Wheat middling	14	Lysine	0.99
Soybean meal	7	Methionine	0.44
Pork oil	3.35		
CaHPO_4_	1.15		
Stone powder	0.4		
NaCl	0.32		
Lysine	0.48		
Premixes^a^	1		
Total	100		

^a^Premix is provided per kg: V_A_ 8,000 IU, V_D_ 1700 IU, V_E_ 13 IU, V_K_ 0.9 mg, VB_1_ 1 mg, VB_2_ 3 mg, VB_6_ 1.3 mg, VB_12_ 0.01 mg; Pantothenic acid 12 mg, Niacin 16mg, Folic acid 3 mg, Fe 55 mg, Cu 25 mg, Zn 35 mg, Mn 60 mg, Se 0.3 mg, I 0.8 mg.

^b^Nutrient levels are measured except for metabolizable energy.

**Table 2:** Composition and nutritional level of CSFBP-fermented feed.

**Ingredients**	**Content (%)**	**Nutritional level^b^**	**Content (%)**
Corn	10	Metabolizable energy (MJ/kg)	8.2
Corn germ meal	3.03	Crude Ash	8.88
Soybean meal	13.8	Crude protein	24.37
Corn bran residue	20.6	Moisture	33.63
Citri sarcodactylis fructus residues	20	Ca	1.13
Rice husk powder	3	Lysine	0.87
Bran	3	P	0.5
H_2_O	23.72	Methionine	0.75
Stone powder	1.41		
Premixes^a^	0.25		
CaHPO_4_	0.46		
NaCl	0.33		
NaHCO_3_	0.1		
Enzyme preparation	0.1		
Compound bacteria	0.1		
Methionine	0.1		
Total	100		

^a^Premix is provided per kg: V_A_ 8,000 IU, V_D_ 1,700 IU, V_E_ 13 IU, V_K_ 0.9 mg, VB_1_ 1 mg, VB_2_ 3 mg, VB_6_ 1.3 mg, VB_12_ 0.01 mg; Pantothenic acid 12 mg, Niacin 16 mg, Folic acid 3 mg, Fe 55 mg, Cu 25 mg, Zn 35 mg, Mn 60 mg, Se 0.3 mg, I 0.8 mg.

^b^Nutrient levels are measured except for metabolizable energy.

### 2.3. Sample collection

At the end of the trial, a 12-h fasting period was imposed for all birds prior to weighing. Subsequently, one bird was randomly chosen from each group, euthanized through cervical dislocation, and dissected for sampling purposes. The breast muscle weight, dressed weight, fully-eviscerated weight, semi-eviscerated weight, and leg muscle weight were measured to determine the slaughter performance. The contents of the cecum, ileum, jejunum, and duodenum of each chicken were collected, frozen in liquid nitrogen (−196°C), and transferred to an ultra-low temperature freezer at −80°C for measuring intestinal enzyme activity, cecal microbiota, and metabolites. The breast muscles of each chicken were likewise flash-frozen in liquid nitrogen (−196°C) and then transferred to an ultra-low temperature freezer set at −80°C for subsequent analysis of amino acid and fatty acid contents.

### 2.4. Slaughter performance

The eviscerated carcass yield, semi-eviscerated carcass yield, thigh yield, slaughter yield, and breast yield were calculated based on the measured live weight, eviscerated carcass weight, semi-eviscerated carcass weight, thigh weight, slaughter weight, and breast weight.

Slaughter yield (%) = Slaughter weight/live weight × 100%

Semi-eviscerated carcass yield (%) = Semi-eviscerated carcass weight/live weight × 100%

Eviscerated carcass yield (%) = Eviscerated carcass weight/live weight × 100%

Thigh yield (%) = Thigh weight/Eviscerated carcass weight × 100%

Breast yield (%) = Breast weight/Eviscerated carcass weight × 100%

### 2.5. Intestinal digestive enzyme activity

The activity of pancreatic protease, lipase, and amylase in the duodenum, jejunum, and ileum were determined using assay kits, according to the manufacturer's instructions (A080-2-2, A454-2-1 and C016-1-1, NanJing JianCheng Bioengineering Institute, Nanjing, China). The unit of pancreatic protease activity was defined as the quantity of enzyme that induces a variation in absorbance of 0.003 per minute per milligram of protein under conditions of pH 8.0 and 37°C. The amylase activity was quantified by determining the quantity of enzyme required to hydrolyze 10 mg of starch within a 30-min duration at 37°C per milligram of protein present in the tissue. The lipase activity was determined by measuring the quantity of enzyme that metabolizes 1 μmol of substrate within a minute per gram of tissue protein at 37°C in the reaction system.

### 2.6. Apparent nutrient utilization

The Automatic Kjeldahl nitrogen analyzer was utilized to measure the nitrogen content. Subsequently, this measurement was employed to determine the crude protein content by multiplying it by a factor of 6.25. Ether extract content was determined by the Soxhlet extraction method. Crude ash content was determined by sintering in a muffle furnace at 550°C. Phosphorus was measured using a spectrophotometer at 400 nm. Calcium was measured titrimetric ally using the potassium permanganate method (18, 19).

### 2.7. Amino acid contents

An appropriate amount of chicken breast sample (around 1 g) was weighed out. 15 mL of 6 mol/L hydrochloric acid solution was added to a digestion tube. The digestion tube was placed into a freezing agent and frozen for 3–5 min. It was then protected with nitrogen gas, and the bottle cap was tightened. The hydrolysis was carried out in a constant-temperature oven at 110°C for 22 h. After removal, it was allowed to cool down to ambient temperature. The digestion tube was opened, and the hydrolysate was filtered into a 50 mL volumetric flask. The digestion tube was rinsed multiple times with a small quantity of water, and it was ultimately diluted with water until the mark was reached. The flask was thoroughly shaken to ensure homogeneity. 1.0 mL of the filtrate was accurately transferred into a 15 mL test tube, and it was evaporated to dryness at 40°C under reduced pressure. Then, it was dissolved with 1.0 mL of pH 2.2 sodium citrate buffer solution, shaken well, and filtered through a 0.22 μm filter membrane. Finally, it was determined using an amino acid automatic analyzer (Hitachi LA8080).

### 2.8. Fatty acid contents

A suitable quantity of chicken breast was measured and approximately 100 mg of caffeic acid was incorporated. A small number of zeolite pieces and 2 mL of 95% ethanol were introduced, then thoroughly combined. 10 mL of hydrochloric acid solution was added and ensured thorough mixing. The flask was submerged in a water bath at 75°C and allowed to hydrolyze for 40 min. The flask was shaken every 10 min to incorporate any particles stuck to the inner wall. Once the hydrolysis was complete, the flask was taken out and allowed to cool down to room temperature. The hydrolyzed sample was mixed well by adding 10 mL of 95% ethanol. The hydrolyzed solution was then transferred from the flask to a separatory funnel, and the flask and stopper were rinsed with 50 mL of a mixture of ether and petroleum ether. The flask was covered, shaken for 5 min, and allowed to settle for 10 min. The extracted ether layer was gathered in a 250 mL flask. These steps were repeated three times to extract the hydrolyzed solution, and finally, the separatory funnel was washed with a mixture of ether and petroleum ether, collecting it in the pre-weighed flask. The flask was placed in a water bath, and the contents were evaporated at 103°C for 2 h until dry. The fat extract was combined with 2 mL of a methanol solution containing 2% sodium hydroxide, and hydrolysis was performed in a water bath at 85°C for 30 min. Next, 3 mL of a methanol solution containing 14% boron trifluoride was introduced, and hydrolysis was continued in the same 85°C-water bath for an additional 30 min. After the water bath, the mixture was allowed to cool to room temperature. Then, 1 mL of n-hexane was added to a centrifuge tube and shaken for 2 min. It was left to stand for 1 h, allowing the layers to separate. From the upper layer, 100 μL of the clear liquid was extracted and diluted to 1 mL using n-hexane. After passing it through a 0.45 μm filter membrane, a gas chromatography-mass spectrometer (Thermo, Trace1310 ISQ) was employed for detection.

### 2.9. 16S rDNA sequencing of the cecum microbiota

The appendix contents preserved in a freezer at −80°C were thawed, and genomic DNA was extracted from them using a DNA extraction kit (Magen D6356-02). The DNA concentration was subsequently assessed by employing agarose gel electrophoresis and NanoDrop2000. To guarantee accurate and efficient amplification, PCR was conducted using specific primers incorporating barcodes designed for the targeted sequencing region, utilizing the extracted genomic DNA as the template (Takara R060B). Following PCR amplification, the amplified products underwent electrophoresis for detection, followed by purification using magnetic beads. These purified products were then employed as templates for a subsequent round of PCR amplification. The resulting products were once again subjected to electrophoresis for detection, followed by purification using magnetic beads. Quantification of the purified products was performed using Qubit. Subsequently, the PCR products, mixed equally based on their concentration, were subjected to library preparation. The library was sequenced on the Illumina NovaSeq6000 sequencing platform to generate the raw data.

The raw data obtained was in FASTQ format. Upon sequencing completion, the cutadapt software was applied to remove the primer sequences from the raw data sequences. Subsequently, DADA2 with the default parameters of QIIME 2 (Quantitative Insights Into Microbial Ecology) was utilized to carry out quality filtering, denoising, merging, and removal of chimeric sequences. This analysis was performed on the paired-end raw data that met the quality criteria, resulting in representative sequences and abundance tables of ASVs. After utilizing the QIIME 2 software package to select representative sequences for each ASV, the aligned representative sequences were annotated with the Silva database (version 138). The Silva database (version 138) was employed for 16S alignment, and ASV clustering and species classification analysis were performed based on the high-quality data. Subsequently, Alpha Diversity and Beta Diversity measures were employed to uncover the variations in species composition within and between the treatment groups. PCoA was used to investigate dissimilarities in community structure among the treatment groups. Finally, statistical methods like LEfSe were employed to analyze taxonomic level-specific differentially abundant microorganisms.

### 2.10. Gut flora metabolome

A measurement of 60 mg of cecal contents was taken and transferred to a 1.5 mL centrifuge tube. Two small steel beads were introduced, and a methanol-water solution (V: V = 1:1) containing L-2-chlorophenylalanine at a concentration of 4 μg/mL (600 μL) was added. The tube was pre-cooled in a −40°C freezer for 2 min, after which the contents were ground using a homogenizer (60 Hz, 2 min). Ultrasound-assisted extraction was performed for 30 min in an ice-water bath, followed by an additional incubation at −40°C for 30 min. The tube was then centrifuged at low temperature (12,000 rpm, 4°C) for 10 min, and 150 μL of the resulting supernatant was carefully transferred to a glass derivatization vial. Moisture was removed from the sample using a centrifugal concentrator. A pyridine solution containing methoxyamine hydrochloride (15 mg/mL) (80 μL) was added to the vial, and the mixture was incubated at 37°C with agitation for 60 min to facilitate the oximation reaction. To the vial, 50 μL of BSTFA derivatization reagent and 20 μL of n-hexane were added. Additionally, 10 internal standards (C8/C9/C10/C12/C14/C16/C18/C20/C22/C24, all dissolved in chloroform) at a volume of 10 μL were included. The reaction was carried out at 70°C for 60 min. After the sample was removed, it was allowed to rest at room temperature for 30 min before proceeding with GC-MS metabolomics analysis.

The experimental setup for chromatography included the following conditions: a DB-5MS capillary column (30 m × 0.25 mm × 0.25 μm, Agilent J&W Scientific, Folsom, CA, USA) was employed, utilizing high purity helium gas (purity ≥ 99.999%) as the carrier gas with a flow rate of 1.0 mL/min. The injection port temperature was maintained at 260°C. A 1 μL injection volume was applied without implementing split injection, and a solvent delay of 4.8 min was observed. The temperature program during the analysis consisted of the following steps: the column oven was initially set at 60°C and maintained for 0.5 min. Subsequently, the temperature was raised at a rate of 8°C/min until reaching 125°C. It was then further increased at the same rate to 210°C, followed by a subsequent increase at a rate of 15°C/min to 270°C. Finally, the temperature was raised at a rate of 20°C/min until reaching 305°C, where it was held steady for 5 min. The following conditions were employed for mass spectrometry analysis: an electron impact ionization source (EI) was utilized with the ion source temperature adjusted to 230°C, while the quadrupole temperature was set at 150°C. The electron energy applied was 70 eV. Full scan mode (SCAN) was chosen as the scan mode, covering a mass scan range of m/z 50–500.

### 2.11. Statistical analysis

The data are expressed as “mean ± standard deviation” and visualized using GraphPad Prism 8.0. Statistical analysis was conducted using SPSS 23.0 software, employing one-way ANOVA with a significance level of *P* < 0.05. Significance was denoted by distinct lowercase letters as superscripts.

## 3. Results

### 3.1. Growth performance

Table 3 illustrates the effects of incorporating FCSF into the diet on the growth performance of birds. After feeding FCSF for 42 days, the FW and ADG of birds were significantly higher than those of T1 (*p* < 0.05). Compared to T1, supplementing FCSF significantly reduced the F/G, ADFI, and Mortality of broiler chickens (*p* < 0.05). Supplementation of the 3% FCSF has the most significant growth advantage.

**Table 3:** The impacts of FCSF on the growth performance of birds.

**Parameters**	**T1**	**T2**	**T3**	**T4**	***P*-value**
IW (kg)	1.48 ± 0.02	1.47 ± 0.02	1.41 ± 0.02	1.46 ± 0.03	0.283
FW (kg)	2.92 ± 0.06^b^	3.19 ± 0.02^a^	3.28 ± 0.04^a^	3.22 ± 0.04^a^	<0.05
F/G	4.60 ± 0.20^a^	3.73 ± 0.06^b^	3.37 ± 0.06^c^	3.61 ± 0.10b^c^	<0.05
ADFI (g)	156.70 ± 0.59^a^	152.23 ± 0.72^b^	149.06 ± 0.98^b^	150.77 ± 1.95^b^	<0.05
ADG (g)	34.40 ± 1.58^c^	40.85 ± 0.74^b^	44.34 ± 0.72^a^	41.87 ± 0.69^ab^	<0.05
Mortality (%)	9.69 ± 1.98^a^	4.53 ± 0.99^b^	4.59 ± 1.43^b^	4.86 ± 1.06^b^	<0.05

^a−*c*^Significant difference in the mean of different letters in a row (*n* = 6, *P* < 0.05). FW, final weight; IW, initial weight; FCR, feed conversion rate; ADFI, average daily feed intake; ADG, average daily gain.

### 3.2. Slaughter performance

Table 4 presents the slaughter performance of birds following the addition of FCSF to their diet. Compared to the control group, supplementing 3% and 5% FCSF significantly improved the semi-eviscerated carcass yield, slaughter yield, and thigh yield of birds (*p* < 0.05). Adding FCSF to the feed significantly improved the eviscerated carcass yield of broiler chickens (*p* < 0.05).

**Table 4:** The impact of FCSF on the slaughter performance of birds (%).

**Parameters^A^**	**T1**	**T2**	**T3**	**T4**	***P*-value**
Slaughter yield	86.90 ± 1.41^b^	88.76 ± 0.38^ab^	90.89 ± 1.48^a^	90.44 ± 0.41^a^	<0.05
Semi-eviscerated carcass yield	80.39 ± 0.59^b^	81.70 ± 0.39^ab^	83.82 ± 1.40^a^	83.99 ± 0.44^a^	<0.05
Eviscerated carcass yield	63.27 ± 0.63^c^	65.90 ± 0.35^b^	66.75 ± 0.57^b^	68.43 ± 0.30^a^	<0.05
Breast yield	17.14 ± 0.42	17.99 ± 0.60	17.29 ± 0.32	17.93 ± 0.28	0.392
Thigh yield	23.69 ± 1.09^b^	21.86 ± 0.82^bc^	24.81 ± 0.64^a^	25.24 ± 0.47^a^	<0.05

^a−*c*^Significant difference in the mean of different letters in a row (*n* = 6, *P* < 0.05).

^A^Calculated as a percentage of live BW. Expressed as a proportion relative to the weight of the eviscerated carcass.

### 3.3. Intestinal digestive enzyme activity

Table 5 presents the activities of intestinal digestive enzymes in broilers following the supplementation of FCSF. There was no significant difference in α-amylase activity in the different intestinal segments of broilers after 42 days of FCSF supplementation (*p* > 0.05). Compared with the control group, feeding 3% and 5% FCSF significantly increased lipase activity in the duodenum and ileum of broiler, and supplementing 3% FCSF significantly increased protease activity in the duodenum of broilers (*p* < 0.05).

**Table 5:** The impact of FCSF on the intestinal digestive enzyme activity of broiler chickens.

**Parameters**	**T1**	**T2**	**T3**	**T4**	***P*-value**
**Duodenum**					
Protease	54.62 ± 4.61^b^	54.46 ± 6.89^b^	93.42 ± 19.57^a^	72.57 ± 4.34^ab^	<0.05
Lipase	32.64 ± 2.52^b^	40.18 ± 3.22^ab^	47.71 ± 3.40^a^	43.09 ± 2.66^a^	<0.05
α-Amylase	227.07 ± 31.56	223.57 ± 26.93	304.75 ± 23.48	297.99 ± 31.40	0.100
**Jejunum**					
Protease	69.10 ± 5.94	70.41 ± 13.12	85.35 ± 14.05	71.96 ± 6.93	0.687
Lipase	59.62 ± 3.10	66.28 ± 6.57	76.01 ± 7.69	66.47 ± 2.37	0.233
α-Amylase	179.09 ± 14.26	177.24 ± 43.21	182.61 ± 8.91	165.60 ± 5.39	0.959
**Ileum**					
Protease	30.08 ± 4.15	30.64 ± 6.87	33.23 ± 5.20	28.91 ± 1.74	0.934
Lipase	27.78 ± 1.22^b^	30.52 ± 1.64^ab^	35.27 ± 1.29^a^	34.59 ± 2.51^a^	<0.05
α-Amylase	162.19 ± 6.47	158.05 ± 16.94	176.56 ± 13.45	155.95 ± 8.62	0.628

^a, b^Significant difference in the mean of different letters in a row (*n* = 6, *P* < 0.05).

### 3.4. Nutrient utilization

Table 6 presents the nutrient utilization in broilers following the supplementation of FCSF. Compared with the control group, the addition of 3% FCSF significantly improved the utilization of total phosphorus, crude ash, crude protein, and dry matter in the feed by broilers (*p* < 0.05). The utilization of crude protein, dry matter, crude ash, and total phosphorus in the feed by broilers was slightly improved after adding 1% and 5% FCSF, and the utilization of calcium and ether extract in the feed by broilers was similar even after adding FCSF.

**Table 6:** The impacts of FCSF on the nutrient utilization of birds.

**Parameters**	**T1**	**T2**	**T3**	**T4**	***P*-value**
Dry matter	69.20 ± 2.14^b^	72.41 ± 1.88^ab^	74.15 ± 3.37^a^	71.44 ± 4.21^ab^	<0.05
Ether extract	79.52 ± 4.69	83.94 ± 1.9^6^	82.07 ± 2.47	79.83 ± 9.96	0.591
Crude protein	49.71 ± 6.29^b^	50.43 ± 6.83^b^	62.30 ± 4.80^a^	61.84 ± 3.37^a^	<0.05
Crude ash	52.37 ± 6.83^b^	54.66 ± 5.33^ab^	62.32 ± 7.01^b^	58.10 ± 6.33^ab^	<0.05
Calcium	52.49 ± 15.81	56.74 ± 9.92	57.99 ± 13.32	59.97 ± 11.87	0.825
Total phosphorus	46.46 ± 4.35^c^	57.95 ± 3.44^ab^	60.48 ± 6.39^a^	50.05 ± 11.37^bc^	<0.05

^a−*c*^Significant difference in the mean of different letters in a row (*n* = 6, *P* < 0.05).

### 3.5. Meat quality

The amino acid and fatty acid contents of bird breast muscles following FCSF supplementation are presented in Tables 7, 8, respectively. We identified 17 amino acids in the breast meat, and found that supplementing with 3% and 5% FCSF reduced the serine content (*p* < 0.05). After supplementing with 1% FCSF, the cystine and tyrosine contents were higher than those in the 3% and 5% FCSF groups (*p* < 0.05).

**Table 7:** The impacts of FCSF on amino acid profiles in birds breast muscle.

**Amino acid^A^**	**T1**	**T2**	**T3**	**T4**	***P*-value**
Aspartic acid	1.90 ± 0.03	1.91 ± 0.03	1.83 ± 0.02	1.86 ± 0.03	0.187
Threonine	0.91 ± 0.02	0.92 ± 0.01	0.87 ± 0.01	0.89 ± 0.01	0.137
Serine	0.76 ± 0.01^a^	0.76 ± 0.02^a^	0.69 ± 0.01^b^	0.70 ± 0.01^b^	<0.05
Glutamic acid	2.91 ± 0.06	2.92 ± 0.05	2.78 ± 0.04	2.85 ± 0.05	0.206
Glycine	0.96 ± 0.10	0.83 ± 0.01	0.80 ± 0.01	0.84 ± 0.04	0.212
Alanine	1.19 ± 0.03	1.16 ± 0.03	1.11 ± 0.02	1.14 ± 0.01	0.111
Cystine	0.15 ± 0.00^ab^	0.16 ± 0.01^a^	0.14 ± 0.01^b^	0.14 ± 0.00^b^	<0.05
Valine	0.99 ± 0.02	0.99 ± 0.01	0.97 ± 0.03	0.99 ± 0.04	0.786
Methionine	0.41 ± 0.03	0.43 ± 0.04	0.42 ± 0.01	0.44 ± 0.02	0.771
Isoleucine	0.91 ± 0.02	0.94 ± 0.01	0.90 ± 0.01	0.92 ± 0.02	0.489
Leucine	1.64 ± 0.03	1.66 ± 0.03	1.60 ± 0.03	1.63 ± 0.03	0.545
Tyrosine	0.65 ± 0.01^ab^	0.67 ± 0.02^a^	0.62 ± 0.01^b^	0.63 ± 0.01^b^	<0.05
Phenylalanine	0.81 ± 0.01	0.81 ± 0.01	0.77 ± 0.01	0.79 ± 0.02	0.175
Lysine	1.81 ± 0.04	1.84 ± 0.03	1.77 ± 0.02	1.80 ± 0.03	0.541
Histidine	0.68 ± 0.04	0.77 ± 0.03	0.76 ± 0.03	0.75 ± 0.03	0.180
Arginine	1.30 ± 0.02	1.28 ± 0.02	1.22 ± 0.01	1.25 ± 0.02	0.101
Proline	0.71 ± 0.04	0.65 ± 0.01	0.67 ± 0.01	0.71 ± 0.03	0.308
TAA	18.68 ± 0.28	18.71 ± 0.33	17.93 ± 0.22	18.32 ± 0.35	0.256
EAA	7.15 ± 0.17	7.35 ± 0.15	7.06 ± 0.10	7.17 ± 0.14	0.527
NEAA	11.53 ± 0.19	11.35 ± 0.18	10.87 ± 0.12	11.15 ± 0.22	0.100
DAA	7.77 ± 0.13	7.64 ± 0.12	7.29 ± 0.08	7.48 ± 0.15	0.073

^a, b^Significant difference in the mean of different letters in a row (*n* = 6, *P* < 0.05).

^A^DAA, delicious amino acids; NEAA, Non-essential amino acids; EAA, essential amino acids; TAA, total amino acids.

**Table 8:** The effects of FCSF on fatty acid content in broiler breast muscle.

**Fatty acid^A^**	**T1**	**T2**	**T3**	**T4**	***P*-value**
C12:0	5.84 ± 1.13	5.28 ± 1.21	4.05 ± 0.57	4.36 ± 0.31	0.483
C14:0	25.37 ± 3.97^b^	36.30 ± 1.94^a^	25.85 ± 2.70^b^	23.02 ± 2.53^b^	<0.05
C14:1	9.73 ± 1.06^b^	13.40 ± 1.39^a^	9.38 ± 0.73^b^	8.87 ± 0.43^b^	<0.05
C15:0	9.31 ± 0.95	10.50 ± 1.41	7.53 ± 0.62	7.78 ± 0.51	0.129
C16:0	1514.99 ± 146.17	1802.82 ± 196.45	1502.33 ± 229.41	1361.20 ± 192.07	0.454
C16:1	86.35 ± 17.15	244.91 ± 64.51	147.32 ± 38.96	96.51 ± 26.72	0.054
C17:0	15.62 ± 1.82	17.24 ± 2.10	14.19 ± 1.48	13.23 ± 0.99	0.369
C18:0	966.03 ± 64.66	750.04 ± 383.93	733.90 ± 76.04	718.42 ± 83.23	0.064
C18:1n9c	1468.94 ± 185.24	2474.56 ± 454.80	1767.96 ± 383.52	1360.49 ± 324.48	0.149
C18:2n6c	1200.28 ± 125.27	1625.18 ± 140.03	1315.46 ± 207.64	1118.88 ± 179.19	0.191
C20:0	28.94 ± 2.18	28.03 ± 4.83	24.12 ± 3.11	22.89 ± 1.26	0.472
C18:3n6	12.26 ± 1.85^b^	16.87 ± 1.53^a^	10.99 ± 0.68^b^	11.28 ± 0.98^b^	0.023
C18:3n3	23.15 ± 3.36^b^	44.09 ± 5.11^a^	30.51 ± 7.50^ab^	22.12 ± 5.11^b^	0.043
C20:1	29.26 ± 2.91^b^	40.23 ± 2.38^a^	25.67 ± 1.69^b^	23.76 ± 3.07^b^	<0.05
C21:0	8.84 ± 0.56	9.87 ± 2.59	7.60 ± 1.29	8.14 ± 0.63	0.738
C20:2	39.98 ± 2.50	40.05 ± 6.53	27.34 ± 2.07	28.70 ± 2.12	0.045
C22:0	30.93 ± 1.65	25.78 ± 6.08	21.71 ± 3.50	22.32 ± 1.68	0.306
C20:3n6	56.85 ± 3.82	48.51 ± 11.28	42.18 ± 5.52	37.01 ± 3.35	0.230
C20:3n3	8.32 ± 0.39	8.48 ± 2.10	6.27 ± 0.81	7.26 ± 0.69	0.541
C22:1n9	85.63 ± 3.50	68.17 ± 15.86	64.97 ± 10.97	70.07 ± 5.70	0.506
C20:4n6	748.30 ± 55.55^a^	385.85 ± 79.31^b^	396.49 ± 66.62^b^	496.23 ± 38.39^b^	<0.05
C23:0	18.30 ± 1.14^a^	11.73 ± 3.10^b^	9.55 ± 1.61^b^	11.21 ± 0.64^b^	<0.05
C22:2	10.05 ± 0.61	11.64 ± 3.23	8.33 ± 1.31	9.01 ± 0.75	0.603
C20:5n3	3.69 ± 0.54	3.38 ± 1.32	2.77 ± 0.93	1.16 ± 0.20	0.203
C24:0	26.16 ± 1.90	22.98 ± 5.42	20.59 ± 3.74	19.00 ± 1.60	0.522
C24:1	32.30 ± 1.54	25.52 ± 6.00	22.37 ± 3.76	21.40 ± 1.42	0.191
C22:6n3	77.46 ± 5.25^a^	41.25 ± 9.67^b^	39.43 ± 6.53^b^	47.69 ± 6.23^b^	<0.05
∑SFA	2650.32 ± 216.34	2720.58 ± 178.42	2371.42 ± 289.77	2211.57 ± 276.13	0.439
∑MUFA	1712.21 ± 206.56	2866.79 ± 497.09	2037.67 ± 410.83	1581.11 ± 352.91	0.117
∑PUFA	2154.06 ± 172.48	2223.33 ± 110.65	1870.91 ± 186.56	1767.50 ± 210.88	0.232
∑ω-3PUFA	112.63 ± 7.26^a^	97.20 ± 9.93^ab^	78.97 ± 2.47^b^	78.24 ± 4.72^b^	<0.05
∑ω-6PUFA	2017.69 ± 169.33	2076.41 ± 107.05	1765.12 ± 177.39	1663.40 ± 200.29	0.278

^a, b^Significant difference in the mean of different letters in a row (*n* = 6, *P* < 0.05).

^A^C18:0, Stearic acid; C20:1, Gadoleic acid; C12:0, Lauric acid; C20:2, Eicosadienoic acid; C20:5n3, Eicosapentaenoic acid; C17:1, Heptadecanoic acid; C18:2n6c, Linoleic acid; C14:1, Myristylic acid; C22:6n3, Docosahexaenoic acid; C20:3n6, Dihomo-y-linolenic acid; C18:3n3, Linolelaidic acid; C15:0, Pentadecylic acid; C22:0, Docosanoic acid; C18:3n6, Alpha linoleic acid; C17:0, Margaric acid; C16:0, Palmitic acid; C14:0, Myristic acid; C18:1n9c, Oleic acid; C20:0, Arachidonic acid; C20:4n6, Arachidonic acid; C21:0, Undecanoic acid; C23:0, Twenty-three carbonic acid; C24:0, Ditetradecanoic acid; C24:1, Ditetradecenoic acid; ∑PUFA: Total polyunsaturated fatty acids; ∑MUFA: Total monounsaturated fatty acids; ∑SFA: Total Saturated Fatty Acids; ∑ω-3 PUFA = (C22:6n3+C20:5n3+C18:3n3); ∑ω-6 PUFA = (C20:4n6+C18:3n6+C20:3n6+C18:2n6c).

Additionally, we detected 27 fatty acids, and observed that supplementing with 1% FCSF significantly increased the contents of C14:0, C14:1, and C20:1 in the breast meat compared with the other experimental groups (*p* < 0.05). Supplementation with FCSF also significantly decreased the contents of C20:4n6 and C23:0 in the breast meat (*p* < 0.05). Furthermore, the content of ∑ω-3PUFA significantly decreased after supplementing with 3% and 5% FCSF (*p* < 0.05).

### 3.6. Gut microbiota

**Figure 1:**
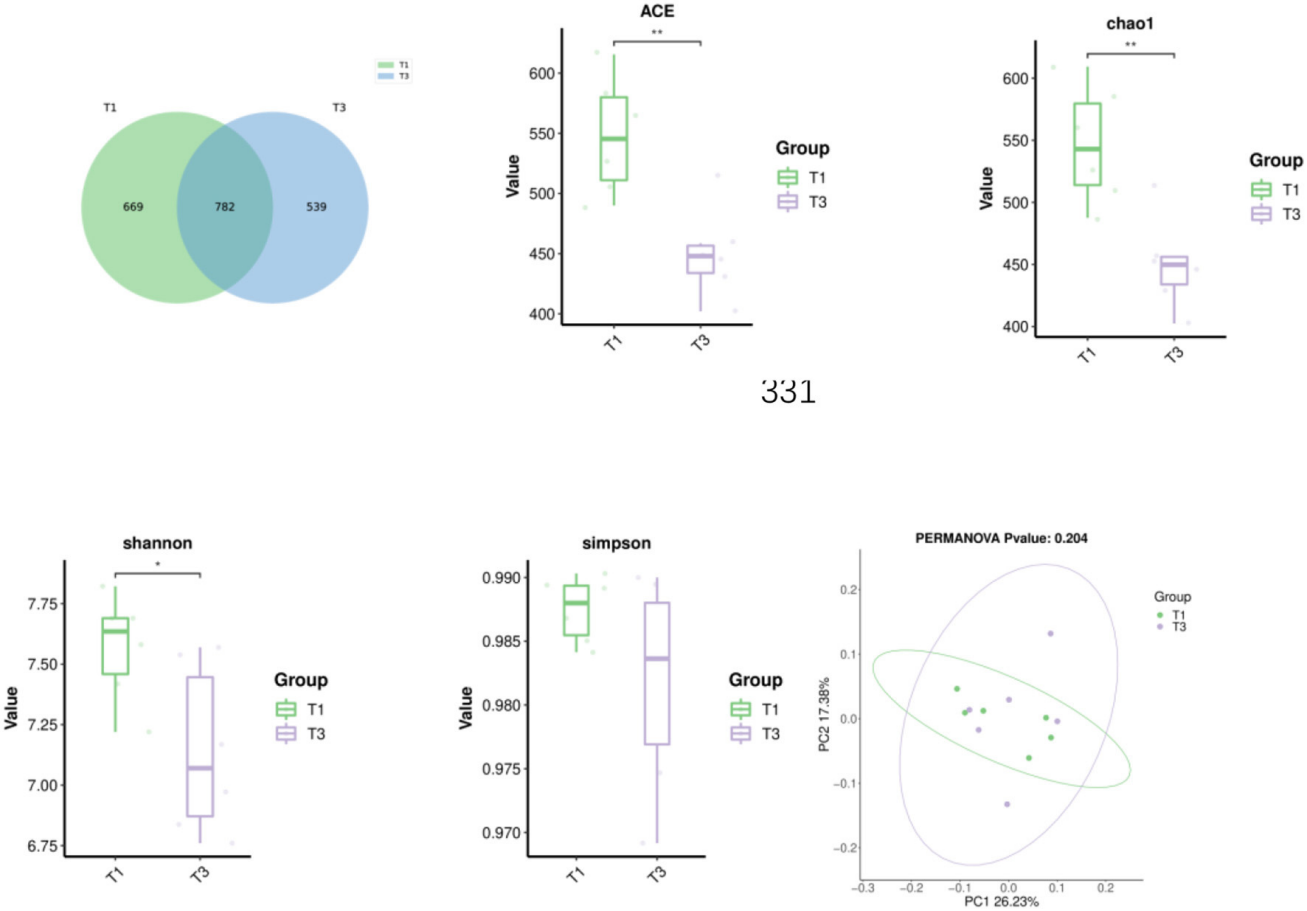
The impacts of incorporating FCSF into the diet on the Venn diagram, β-diversity, and α-diversity of cecal microorganisms in broilers was assessed. Principal Coordinate Analysis (PCoA) was conducted using weighted UniFrac distances derived from the ASV abundance matrix. The presence of ^*^indicates a significant distinction between the two groups (*p < 0.05, **p < 0.01).

As illustrated in Figure 1, the Venn diagram, β-diversity, and α-diversity of cecal microbiota from broiler chickens were examined. A total of 1990 ASVs were detected in the cecal contents, with 782 ASVs shared between the two groups. T1 and T3 had 669 and 539 unique ASVs, respectively. Supplementation with FCSF significantly reduced the ACE, chao1, and Shannon (*p* < 0.05) indices of the cecal microbiota in broiler chickens compared to the control group. PCoA analysis revealed a 43.61% difference between the two principal coordinates. PC1 and PC2 explained 26.23% and 17.38% of the variance, respectively (*p* > 0.05).

**Figure 2:**
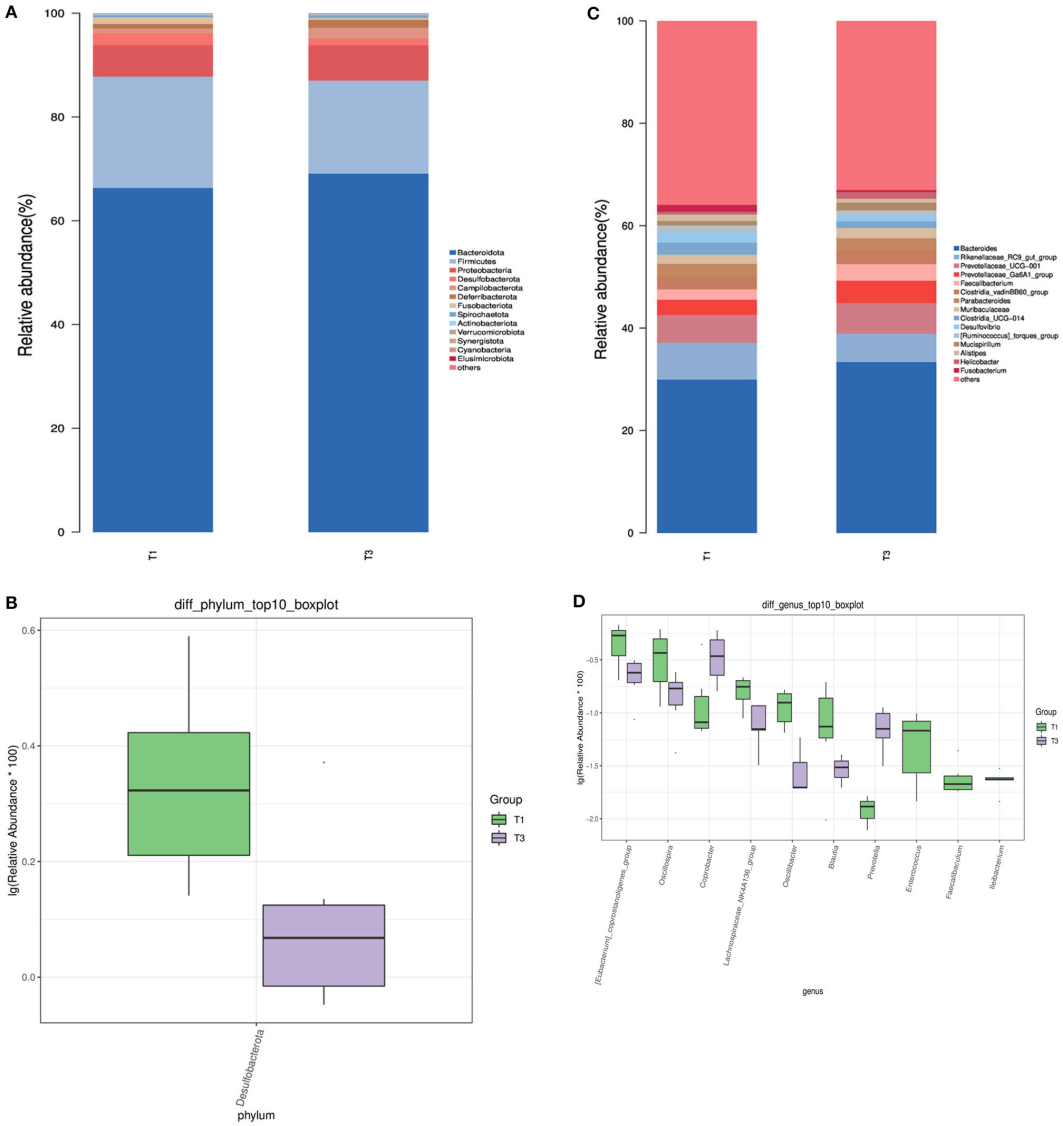
The characterization of microbial composition and identification of distinct species in the cecal microbiota of birds. **(A, C)** Representation of bacterial community composition at the phylum and genus levels, respectively. **(B, D)** Identification of microbial taxa exhibiting significant variations at the phylum and genus levels.

We analyzed the microbial composition of the top 15 taxa at the phylum level (Figure 2A) and found that the main microbial groups in the cecum of birds were *Bacteroidota* and *Firmicutes* (66.32 vs. 69.08%, 21.48 vs. 17.39%, *p* > 0.05). The addition of FCSF decreased the abundance of *Desulfobacterota* in the cecum of broiler chickens (2.31% vs. 1.30%, Figure 2B, *p* < 0.05).

At the genus level, we also analyzed the top 15 taxa (Figure 2C). The dominant species between the two groups included *Prevotellaceae_Ga6A1_group, Prevotellaceae_UCG-001, Rikenellaceae_RC9_gut_group, Bacteroides*, and *Faecalibacterium*. As shown in Figure 2D, there were 10 significantly different species at the genus level, among which the supplementation of FCSF reduced the abundance of *[Eubacterium]_coprostanoligenes_group, Oscillibacter, Lachnospiraceae_NK4A136_group, Oscillospira*, and *Blautia* in the cecum of birds, while increasing the abundance of *Coprobacter* and *Prevotella* (*p* < 0.05). In addition, we also found *Faecalibaculum, Ileibacterium*, and *[Eubacterium]_coprostanoligenes_group* to be unique to the control group.

**Figure 3:**
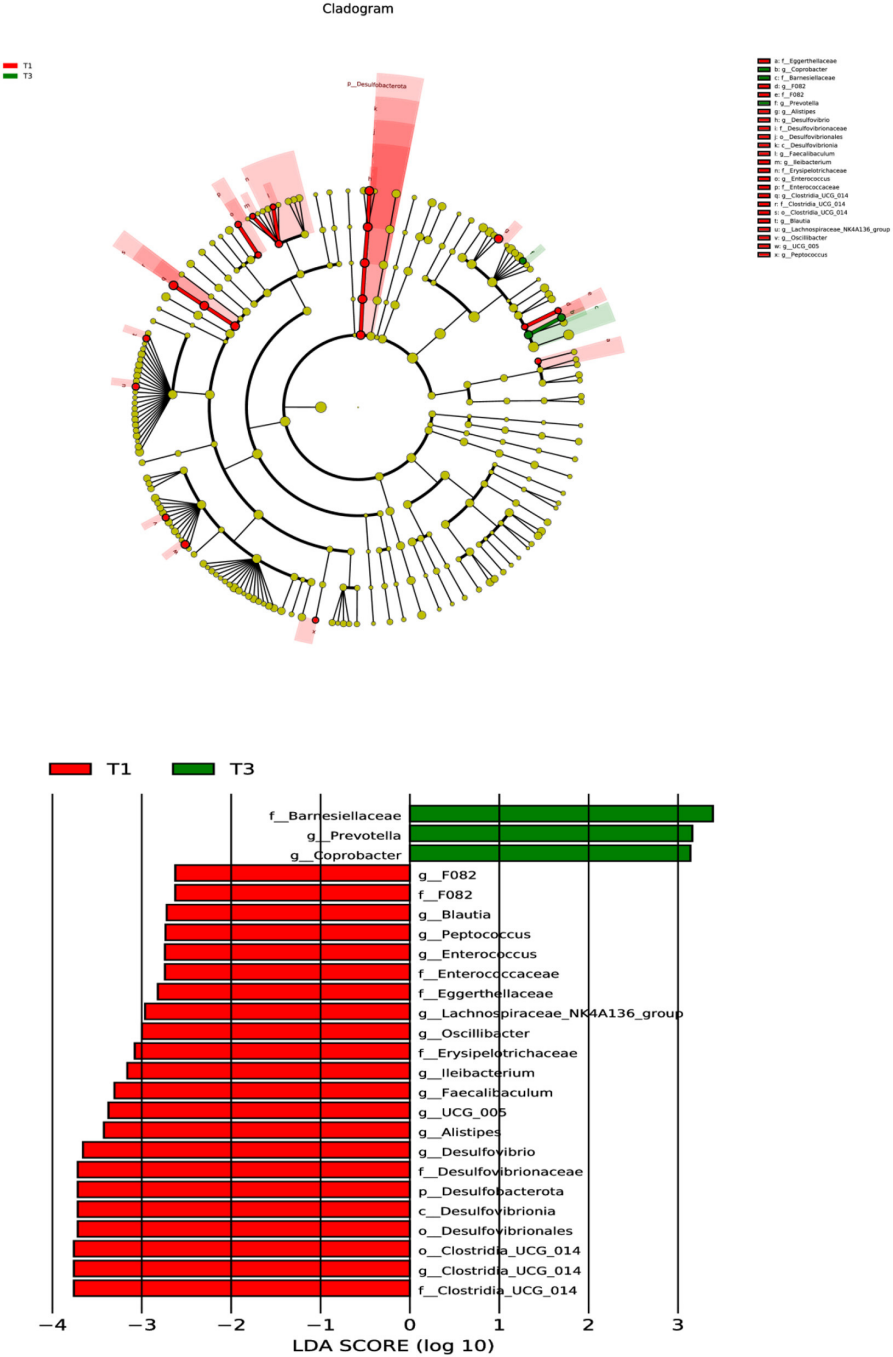
The microbial compositions of the two groups were characterized using LEfSe and LDA analyses, utilizing ASVs as the basis for the analysis (log LDA >3.0; *n* = 6).

A subsequent analysis using LEfSe was conducted on the two groups (Figure 3), revealing a notable dissimilarity in the classification of the T1 and T3 groups. The identified bacterial biomarkers may have potential value with LDA >3 and *P* < 0.05. After supplementing with FCSF, only three potentially valuable biomarkers were found, which were at the Family level of *Barnesiellaceae* and at the Genus level of *Prevotella* and *Coprobacter*. However, a total of 13 potentially valuable biomarkers were found inT1, which were at the Phylum level of *Desulfobacterota*, the Class level of *Desulfovibrionia* and the Order level of *Clostridia_UCG_014* and *Desulfovibrionales*. At the Family level, they were *Erysipelotrichaceae, Desulfovibrionaceae*, and *Clostridia_UCG_014*, and at the Genus level, they were *Clostridia_UCG_014, Desulfovibrio, Alistipes, UCG_005, Faecalibaculum*, and *Ileibacterium*.

### 3.7. Intestinal flora metabolome

**Figure 4:**
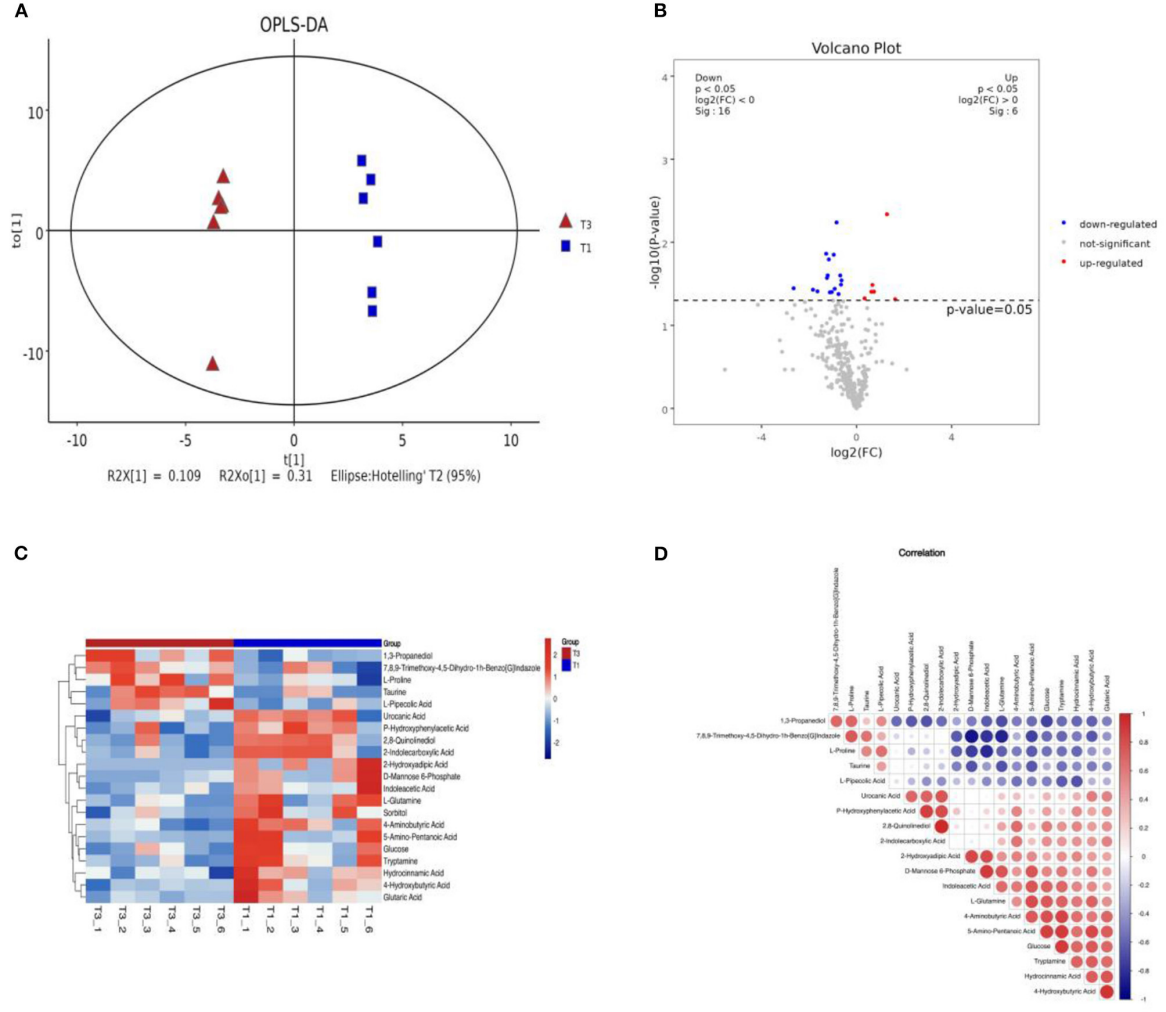
The effects of FCSF on metabolites in chicken cecum contents. **(A)** OPLS-DA analysis of metabolites in chicken cecum contents between two groups. **(B)** A volcano plot was generated to visualize the differential metabolites in chicken cecum contents between the two groups. In this plot, significantly upregulated metabolites are represented by red dots, significantly downregulated metabolites are denoted by blue dots, and non-significant metabolites are depicted as gray dots. **(C)** Hierarchical clustering analysis heatmap of metabolites in chicken cecum contents between two groups, with sample names on the x-axis and differential metabolites on the y-axis, with colors ranging from blue to red indicating low to high expression levels of metabolites, with redder indicating higher expression levels of differential metabolites. **(D)** Correlation analysis was conducted to examine the relationship between metabolites in chicken cecum contents among the two groups. In this analysis, positive correlation is represented by red, negative correlation is indicated by blue, and larger dots signify higher correlation coefficients between the two variables.

We analyzed the differences in metabolites in the cecal contents of broiler chickens using non-targeted metabolomic analysis with GC/MS. The OPLS-DA analysis of the cecal metabolites in Figure 4A indicated a clear distinction between the T1 and T3 groups after 42 days of FCSF supplementation. Figure 4B provides a visual representation of the overall distribution of inter-group metabolic differences. Compared with the T1 group, the T3 group showed upregulation of six metabolites and downregulation of sixteen metabolites. To visually represent the association between samples and the variations in metabolite expression across different samples, hierarchical clustering was conducted on the expression levels of all significantly different metabolites and the top 50 metabolites with the highest VIP (Variable Importance in the Projection) values. The results are depicted in Figure 4C, illustrating the clustering patterns. The top 50 significantly different metabolites in the cecal microbiota of the T3 group compared to the T1 group were D-Mannose 6-Phosphate, L-Glutamine, 2,8-Quinolinediol, Taurine, 1,3-Propanedio, 5-Amino-Pentanoic Acid, Glucose, 4-Hydroxybutyric Acid, Indoleacetic Acid, 2-Hydroxyadipic Acid, Tryptamine, Urocanic Acid, 4-Aminobutyric Acid, 2-Indolecarboxylic Acid, P-Hydroxyphenylacetic Acid, 7,8,9-Trimethoxy-4,5-Dihydro-1h-Benzo[G]Indazole Glutaric Acid, L-Proline, Hydrocinnamic Acid L-Pipecolic, Acid Sorbitol.

Correlation analysis plays a crucial role in quantifying the extent of association among distinct metabolites, facilitating a deeper comprehension of their interrelationships during biological state changes. In this study, we employed the Pearson correlation coefficient, a widely used measure of linear correlation, to evaluate the relationship between pairs of metabolites. Positive correlations are depicted in red, while negative correlations are represented in blue. The size of each circle corresponds to the strength of the correlation coefficient between the respective variables. To explore these relationships, correlation analysis was performed on the top 20 metabolites sorted by VIP, as depicted in Figure 4D.

**Figure 5:**
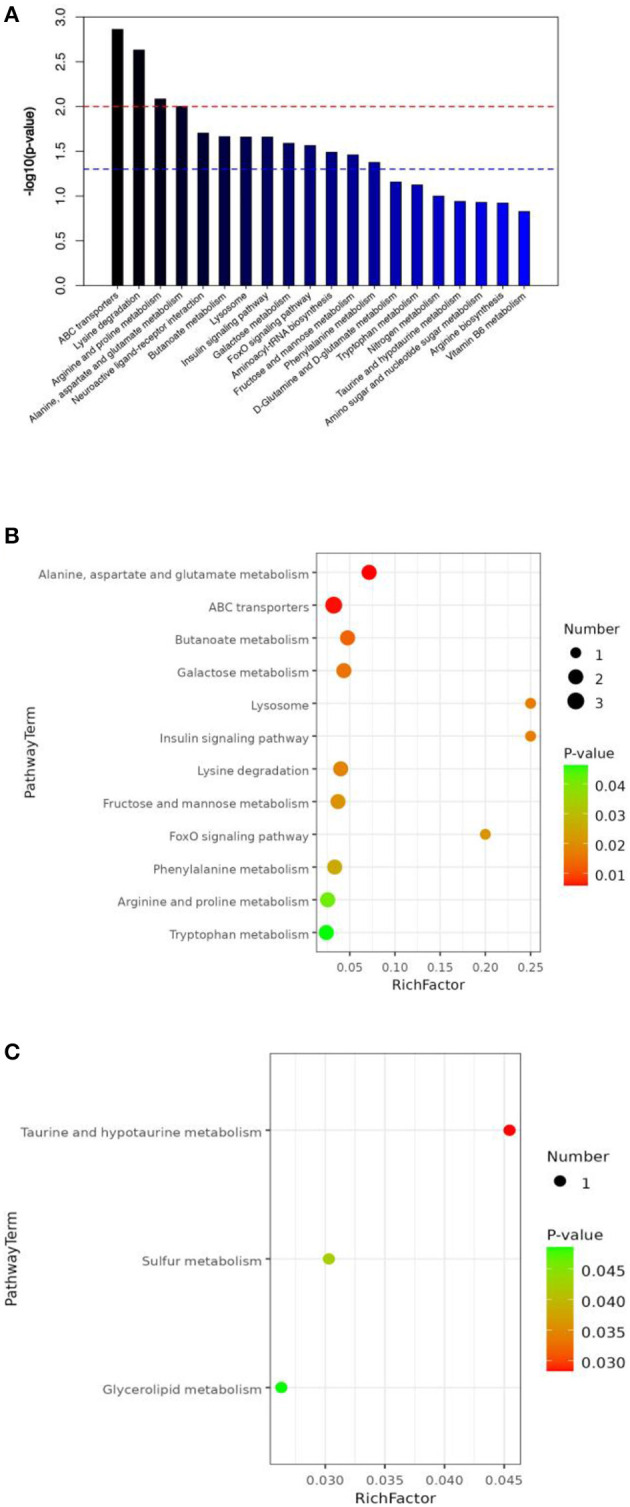
The effect of FCSF supplementation on the KEGG pathway enrichment of the cecal content metabolites in broiler chickens. **(A)** KEGG pathway enrichment (top 20) of cecal metabolites. The red line corresponds to a *p*-value of 0.01, while the blue line corresponds to a *p*-value of 0.05. Signaling pathways represented by bars above the blue line are considered significant. **(B)** Downregulated differentially expressed metabolites (*p* < 0.05). **(C)** Upregulated differentially expressed metabolites (*p* < 0.05).

All metabolites from the T1 and T3 groups were imported into KEGG and pathway enrichment was performed to determine the metabolic pathways in which all metabolites participate. Figure 5A shows the top 20 metabolic pathways enriched by all intestinal metabolites, including lysine degradation, ABC transporters, arginine and proline metabolism, neuroactive ligand-receptor interaction, alanine, aspartate and glutamate metabolism, butanoate metabolism, lysosome, insulin signaling pathway, galactose metabolism, FoxO signaling pathway, and aminoacyl-tRNA biosynthesis.

T1 and T3 groups were compared to identify differential metabolites, and pathway enrichment was performed to determine key metabolic pathways associated with differential metabolites. Bubble plots (Figures 5B, C) were used to show the metabolic pathways enriched with significantly different metabolites. After supplementation with FCSF, a total of 12 metabolic pathways were downregulated in the chicken cecal contents, including tryptophan metabolism, phenylalanine metabolism, fructose, arginine and proline metabolism, galactose metabolism, lysosome, ABC transporters, lysine degradation, insulin signaling pathway, alanine, aspartate and glutamate metabolism, butanoate metabolism, and mannose metabolism, and FoxO signaling pathway (Figure 5B). There were 3 upregulated metabolic pathways, including Glycerolipid metabolism, Sulfur metabolism, and Taurine and hypotaurine metabolism (Figure 5C).

### **3.8. Growth performance-microbiome-metabolome correlation anal**ysis

**Figure 6:**
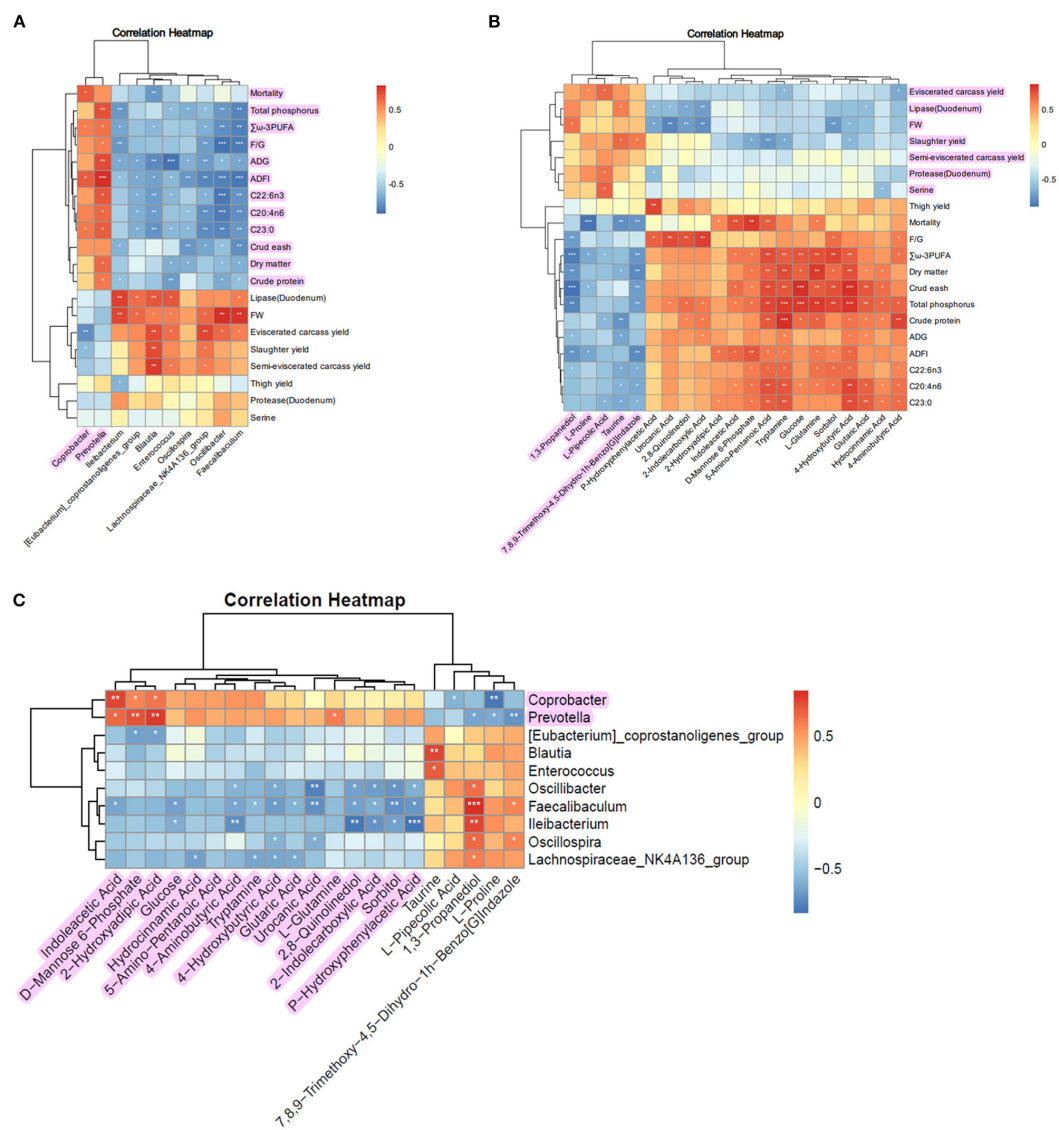
Correlation analysis of growth performance, microbiome, and metabolome in broiler chickens supplemented with FCSF. **(A)** The heatmap displays the correlation analysis between the distinct microbial communities in the cecum and the growth indicators. **(B)** Heatmap illustrating the correlation analysis between differential metabolites in the cecum microbiota and growth indicators. **(C)** The heatmap visually represents the correlation analysis between distinct microbial communities and diverse metabolites in the cecum. The *, **, and *** symbols indicate the values of *P* < 0.05, *P* < 0.01, and *P* < 0.001, respectively.

The Pearson correlation analysis between differentially abundant gut microbiota at the genus level and other chicken indicators yielded an associated heatmap (Figure 6A). In the heatmap, red squares indicate positive correlations, with darker shades of red indicating higher correlation coefficients. Blue squares represent negative correlations, with darker shades of blue indicating higher correlation coefficients. The results show that the upregulated gut microbiota, Coprobacter and Prevotella, after supplementing FCSF, exhibited positive correlations with the metabolites highlighted in purple in Figure 6A, while showing negative correlations with other metabolites.

The Pearson correlation analysis between differentially abundant metabolites in the differential metabolic pathways within the gut and other chicken indicators yielded a heatmap (Figure 6B). The results show that Taurine, 1,3-Propanediol, 7,8,9-Trimethoxy-4,5-Dihydro-1h-Benzo[G]Indazole, L-Proline, and L-Pipecolic Acid exhibited positive correlations (indicated by purple marks in the figure) with indicators such as Eviscerated carcass yield, Lipase (Duodenum), FW, Slaughter yield, Semi-eviscerated carcass yield, Protease (Duodenum), and Serine. These metabolites showed negative correlations with the remaining indicators.

To enhance the visual comprehension of the association between gut microbiota and their corresponding metabolites, we conducted Pearson correlation analysis to examine the correlation between the metabolites exhibiting differential abundance and the differentially abundant microbiota at the genus level. The result is presented in the heatmap (Figure 6C). The findings indicate that the upregulated gut microbiota Coprobacter and Prevotella, following the supplementation of FCSF, exhibited positive correlations (indicated by purple highlights in Figure 6C) with specific metabolites. However, they also showed negative correlations with the remaining metabolites.

## 4. Discussion

Research findings indicate that the incorporation of probiotics into fermented feed exerts favorable impacts on animal physiology, enhancing nutrient digestion and assimilation while minimizing feed resource wastage (20, 21). Research has indicated that supplementing fermented feed can increase the final weight of piglets on day 42, as well as increase their ADG and ADFI, while decreasing their feed conversion ratio (F/G) (22). In another study, supplementing with 3 g/kg of *Lichen Bacillus* fermented product enhanced the body weight and average weight of birds compared to the control group (23). Our previous research also found that supplementing with 5% fermented medicinal herb residues enhanced the FW and ADG of broilers, while decreased their feed to meat ratio (24). Consistent with previous researchers, supplementing with a certain amount of FCSF significantly improved the FW and ADG of broilers, while significantly reduced their F/G, ADFI, and mortality. The evaluation of broiler carcass quality involves considering various parameters, among which slaughter performance holds significant importance. Indicators such as the evisceration rate and semi-evisceration rate directly reflect the slaughter performance and provide valuable insights into broiler carcass quality assessment (25, 26). The research shown that supplementing the diet with fermented Ginkgo biloba leaves at a rate of 3.5 and 4.5 g/kg resulted in a higher percentage of eviscerated yield, along with greater proportions of breast and thigh muscle (27). Our research has revealed that feeding FCSF can enhance the slaughter yield, thigh yield, eviscerated carcass yield, and semi-eviscerated carcass yield of broiler chickens, thereby improving their meat production performance.

Beneficial microorganisms in fermented feed can produce various beneficial substances such as probiotics, organic acids, enzymes, etc. These substances can stimulate the growth and differentiation of intestinal mucosal cells and promote the synthesis and secretion of gut digestive enzymes (28, 29). The function of intestinal digestive enzymes is not only beneficial for the digestion and absorption of feed ingredients by the organism, but also helps to maintain a stable environment in the intestine, prevent bacterial growth, and protect intestinal health. Studies have revealed that the inclusion of fermented soybean meal in the diet of starter broilers leads to a notable enhancement in the activities of protease, lipase, and trypsin in the intestinal contents. Moreover, it also promotes the protease activity specifically in grower broilers (30). Studies have demonstrated that the addition of fermented Ginkgo biloba leaves to the diet of broilers results in elevated amylase and trypsin activity in the duodenum and jejunum. Furthermore, it enhances pancreatic protease activity and lipase activity in the jejunal and ileal regions, leading to improved intestinal digestive function in broilers (31). Our study found that feeding FCSF improved the activity of protease and lipase in the duodenum, as well as the ileal lipase activity of broilers. This may be due to the beneficial microorganisms in FCSF and the metabolic products produced during fermentation promoting the secretion of digestive enzymes. Our study has shown that supplementing FCSF can increase the utilization rate of nutrients in broiler chickens, with the most effective addition level being 3%. Previous research has demonstrated that enzymes secreted by probiotics can break down macromolecules in feed, thereby promoting digestion and absorption in broiler chickens. The higher the activity of digestive enzymes in the chicken's intestine, the stronger their feed utilization and absorption capacity (32, 33). Additionally, fermented feed has been shown to elevate the content of small peptides in the feed by approximately 62%, and the proportion of small peptides increases as the fermentation time is prolonged (30, 34). The increase in nutrient utilization in broiler chickens due to FCSF supplementation may be attributed to the partial degradation of macromolecules in fermented feed into smaller molecules during the fermentation process, which is beneficial for digestion and absorption in poultry (35).

Amino acids serve as the fundamental constituents of proteins, whereas fatty acids constitute the primary components of fats. By measuring the amino acid and fatty acid content in chicken breast meat, its nutritional value can be evaluated (36, 37). After supplementing FCSF, we detected a total of 17 amino acids and 27 fatty acids in the muscles of broiler chickens. Many of these amino acids and fatty acids showed no significant differences. However, it is worth noting that the supplementation of FCSF led to a decrease in the levels of certain non-essential amino acids and Σω-3 polyunsaturated fatty acids in the muscle tissue. This is contrary to some studies, which show that feeding fermented feed can increase the amino acid profile and fatty acid profile of finishing pigs (38, 39) and broiler chickens (40, 41), leading to an improvement of meat flavor and quality. We hypothesize that these variations could be attributed to disparities in experimental subjects, fermentation substrates, and strains. Our research found that supplementing FCSF did not have a significant beneficial effect on the amino acid and fatty acid content in the muscles of broiler chickens. However, additional investigation is warranted to uncover the precise mechanism through which supplementing FCSF modifies the amino acid and fatty acid profiles in birds.

Based on the evaluation of growth indicators in broiler chickens, we conducted an analysis of the gut microbiota diversity and microbial metabolites in the group supplemented with 3% FCSF and the control group. We found that supplementation with FCSF resulted in decreased ACE, Chao1, and Shannon indices in the gut of broiler chickens, leading to reduced microbial diversity. This decrease may be attributed to a reduction in the abundance and diversity of harmful bacteria in the chicken gut following FCSF supplementation, thus promoting intestinal health. This maintained a healthy ecological level of intestinal development, facilitating better digestion, absorption, and transportation functions. At the phylum level, the supplementation of FCSF resulted in a decrease in the abundance of *Desulfobacterota* in the cecum of birds. Studies have shown an increased level of *Desulfobacterota* in patients with diabetic retinopathy, and the LPS produced by *Desulfobacterota* can cause inflammatory damage, disrupt metabolic balance, inhibit endotoxin tolerance, and contribute to the occurrence of retinal lesions (42, 43). At the genus level, the abundance of *Coprobacter* and *Prevotella* improved. *Coprobacter* is a type of bacteria found in the intestines, primarily in healthy individuals. They are part of the gut microbiota and contribute to maintaining gut health. On the other hand, *Prevotella* generally plays a crucial role in the breakdown and digestion of dietary sources abundant in fiber. *Prevotella* can break down cellulose and other complex carbohydrates, producing beneficial short-chain fatty acids such as propionic acid, acetic acid, and butyric acid. These short-chain fatty acids provide energy to intestinal cells and help maintain intestinal health (44–46). Additionally, *Prevotella* is also involved in regulating the function of the intestinal immune system (47). Our study also revealed that the microbial composition in the cecum of the control group of broiler chickens showed the presence of *Faecalibaculum, Ileibacterium*, and *[Eubacterium]_coprostanoligenes_group* at the genus level, while these bacteria were not found in the cecum of broiler chickens supplemented with FCSF. This could be attributed to the effects of probiotics present in FCSF, which establish themselves in the intestines and consequently alter the microbial composition, by inhibiting the growth of certain microorganisms. In groups T1 and T3, there were significant differences observed in 13 and 3 potential biomarkers, respectively. The roles of these biomarkers in the intestinal development of broiler chickens require further investigation. In summary, the addition of FCSF as a supplement may contribute to the modulation of intestinal health by enhancing the population of beneficial bacteria associated with promoting intestinal wellbeing while simultaneously reducing the presence of detrimental bacteria.

Fermented feed can alter microbial metabolites by selectively modulating different metabolic pathways in the gut microbiota. These metabolites can be absorbed by intestinal epithelial cells or transported to the liver through the portal vein, thereby influencing host physiology (48, 49). In this experiment, non-targeted metabolomic analysis was performed on the T1 and T3 groups, and it was observed that the addition of FCSF resulted in the downregulation of differential metabolic pathways primarily related to amino acid metabolism. This finding may help explain the potential impact on the amino acid content in bird breast meat. Additionally, upregulated differential metabolic pathways included sulfur metabolism, taurine and hypotaurine metabolism, and glycerolipid metabolism. Taurine is a highly prevalent amino acid found abundantly in animal tissues and an increasing body of literature suggests that taurine can prevent or treat obesity and metabolic disorders (50). Taurine is considered as a cell-protective molecule because it can maintain normal electron transport chain function, preserve glutathione stores, enhance membrane stability, regulate lipid metabolism, strengthen antioxidant responses, alleviate inflammation, and modulate cellular calcium homeostasis (51, 52). Research has found that taurine can improve muscle loss in broiler chickens under chronic heat stress by inhibiting PERK signaling and reversing cell apoptosis induced by endoplasmic reticulum stress (53). Increased sulfur metabolism can provide benefits in terms of protein synthesis, antioxidant protection, detoxification, immune regulation, and nutrient cycling. These benefits help maintain cellular health, enhance immune function, and promote overall physiological health (54). Increased glycerolipid metabolism can provide benefits in terms of energy storage, cellular structure and function, metabolic regulation, signal transduction, and nutrient absorption and transport. These benefits help maintaining normal physiological functions and promote overall health (55, 56). Our research findings indicate that supplementing FCSF leads to upregulation of beneficial metabolic pathways, including taurine and hypotaurine metabolism, glycerolipid metabolism, and sulfur metabolism in broiler chickens. These findings suggest that these metabolic pathways are advantageous for the chickens.

Due to the ability of fermented feed to positively alter the intestinal microbiota and stimulate different metabolic pathways in the microbiota, resulting in variations in metabolic profiles, it can improve the overall health of the host. In our study, we investigated the relationship between broiler chicken growth performance, microbiota, and metabolites through pearson correlation analysis. The results showed that the upregulated microbiota, including *coprobacter* and *prevotella*, after supplementing FCSF, exhibited positive correlations with certain indicators of growth performance, nutrient utilization, and fatty acid content. Moreover, the differentially abundant metabolites in the gut showed positive correlations with various chicken performance indicators, such as eviscerated carcass yield, lipase (duodenum), FW, slaughter yield, semi-eviscerated carcass yield, protease (duodenum), and serine. Additionally, we observed that the upregulated gut microbiota, *coprobacter* and *prevotella*, were positively correlated with the metabolites highlighted in purple in Figure 6C. These findings provide insights into how FCSF modulates broiler chicken performance, but additional research is necessary to uncover the underlying mechanisms involved in this process.

## 5. Conclusions

In conclusion, the results of this study demonstrate that supplementing FCSF in broiler chicken diets improves FW and ADG, while reducing F/G, ADFI, and mortality. Additionally, the supplementation of FCSF enhances the meat production performance of broiler chickens, including slaughter yield, semi-eviscerated carcass yield, eviscerated carcass yield, and thigh yield. Additionally, supplementing FCSF can also enhance the activity of protease and lipase in the duodenum of broiler chickens, as well as the activity of ileal lipase, thereby improving the utilization of dry matter, crude protein, crude ash, and phosphorus in the feed, increasing the digestion and absorption of nutrients. A better advantage is observed at a supplementation level of 3%. Furthermore, supplementing 3% FCSF can alter the diversity of cecal microbiota and the metabolic products of cecal microbiota, improving intestinal microbial balance and enhancing gut health.

## Data availability statement

The datasets presented in this study can be found in online repositories. The names of the repository/repositories and accession number(s) can be found below: https://www.ncbi.nlm.nih.gov/bioproject; PRJNA940200.

## Ethics statement

The experimental protocols were approved by the Animal Ethics Committee of the Leshan Academy of Agriculture Sciences (LSNK. No20200701).

## Author contributions

Conceptualization: XC and HZ. Methodology: XZho and HZ. Formal analyze: XZho, YJ, SL, LK, JD, CY, and JZ. Writing: XZho. Supervision: XZha, LJ, and XC. Funding acquisition: XC. All authors have read and approved the final manuscript.
